# Human volunteer study of the decontamination of chemically contaminated hair and the consequences for systemic exposure

**DOI:** 10.1038/s41598-020-77930-1

**Published:** 2020-11-30

**Authors:** Samuel Collins, Thomas James, Felicity Southworth, Louise Davidson, Natalie Williams, Emily Orchard, Tim Marczylo, Richard Amlôt

**Affiliations:** 1 Chemical and Environmental Effects Department, Centre for Radiation, Chemicals and Environmental Hazards, Public Health England, Didcot, Oxfordshire UK; 2 Toxicology Department, Centre for Radiation, Chemical and Environmental Hazards, Public Health England, Didcot, Oxfordshire UK; 3 Behavioural Science Team, Emergency Response Department Science and Technology, Public Health England, Porton Down, UK

**Keywords:** Public health, Analytical biochemistry

## Abstract

The decontamination of exposed persons is a priority following the release of toxic chemicals. Efficacious decontamination reduces the risk of harm to those directly affected and prevents the uncontrolled spread of contamination. Human studies examining the effectiveness of emergency decontamination procedures have primarily focused on decontaminating skin, with few examining the decontamination of hair and scalp. We report the outcome of two studies designed to evaluate the efficacy of current United Kingdom (UK) improvised, interim and specialist mass casualty decontamination protocols when conducted in sequence. Decontamination efficacy was evaluated using two chemical simulants, methyl salicylate (MeS) and benzyl salicylate (BeS) applied to and recovered from the hair of volunteers. Twenty-four-hour urinary MeS and BeS were measured as a surrogate for systemic bioavailability. Current UK decontamination methods performed in sequence were partially effective at removing MeS and BeS from hair and underlying scalp. BeS and MeS levels in urine indicated that decontamination had no significant effect on systemic exposure raising important considerations with respect to the speed of decontamination. The decontamination of hair may therefore be challenging for first responders, requiring careful management of exposed persons following decontamination. Further work to extend these studies is required with a broader range of chemical simulants, a larger group of volunteers and at different intervention times.

## Introduction

The decontamination of exposed persons, defined as the timely removal of contaminants that may be on or near to body surfaces^1^, is a priority following the accidental or deliberate release of toxic chemicals. Timely, effective decontamination reduces the risk of harm to those directly affected and prevents any potential escalation of an incident through the uncontrolled spread of contamination.

To develop clear and actionable guidance for first responders, acute healthcare receivers and the public, decontamination interventions should be evidence-based. Traditionally this evidence has been generated by evaluating decontamination interventions using in vitro skin diffusion models^2–10^ or using human volunteers exposed to non-toxic chemical simulants that mimic the properties of chemical agents of concern^10–13^. To date most studies have evaluated the removal of chemical simulants from the skin only. Outputs of these studies have been informative and have led to changes to both UK and US emergency decontamination protocols for chemical incidents^14,15^. However, few studies have examined the effectiveness of current decontamination interventions in removing chemicals from hair^7,16–20^ and even fewer have done so with human volunteers^10,14,21^.

Compared to clothed skin, hair and scalp skin are relatively unprotected areas of the body and are likely to be significant sites of exposure during and after a chemical incident. Whilst hair could act as a protective barrier for the scalp^7,14^, certain chemicals have been demonstrated to diffuse rapidly through hair sebum to the follicles from which they can be absorbed^17,22^. Furthermore, evidence to suggest hair can bind chemical contaminants reducing decontamination efficacy^23–25^ and then rapidly release them back into the surrounding environment^26^ could have important implications for the management of contaminated hair following a chemical incident.

In vitro studies of the efficacy of hair decontamination following exposure to VX^16^ and the sulphur mustard simulants methyl salicylate and 2-chloroethyl ethyl sulphide^19^ revealed that showering alone was the least effective decontamination protocol, whereas the application of Fullers Earth (FE) or Reactive Skin Decontamination Lotion (RSDL) prior to showering substantially improved decontamination efficacy up to 45 min post exposure. The same experiments also showed significant persistence of VX and methyl salicylate (MeS) in hair post-decontamination.

The decontamination of hair is therefore an important knowledge gap that requires further examination. This study aimed to evaluate the effectiveness of current United Kingdom (UK) improvised, interim and specialist mass-casualty decontamination methods when conducted in sequence in removing two chemical simulants, methyl salicylate (simulant for sulphur mustard^27^) and benzyl salicylate (BeS, a recently identified simulant for less volatile, more persistent toxic industrial chemicals^28^) from the hair of human volunteers. Analysis of the two simulants excreted in volunteers’ urine over 24 h provided a measure of systemic simulant exposure.

## Results

### Study 1

Video reviews and detailed observations by the study staff confirmed that all participants adhered to the protocols as described.

**Figure 1:**
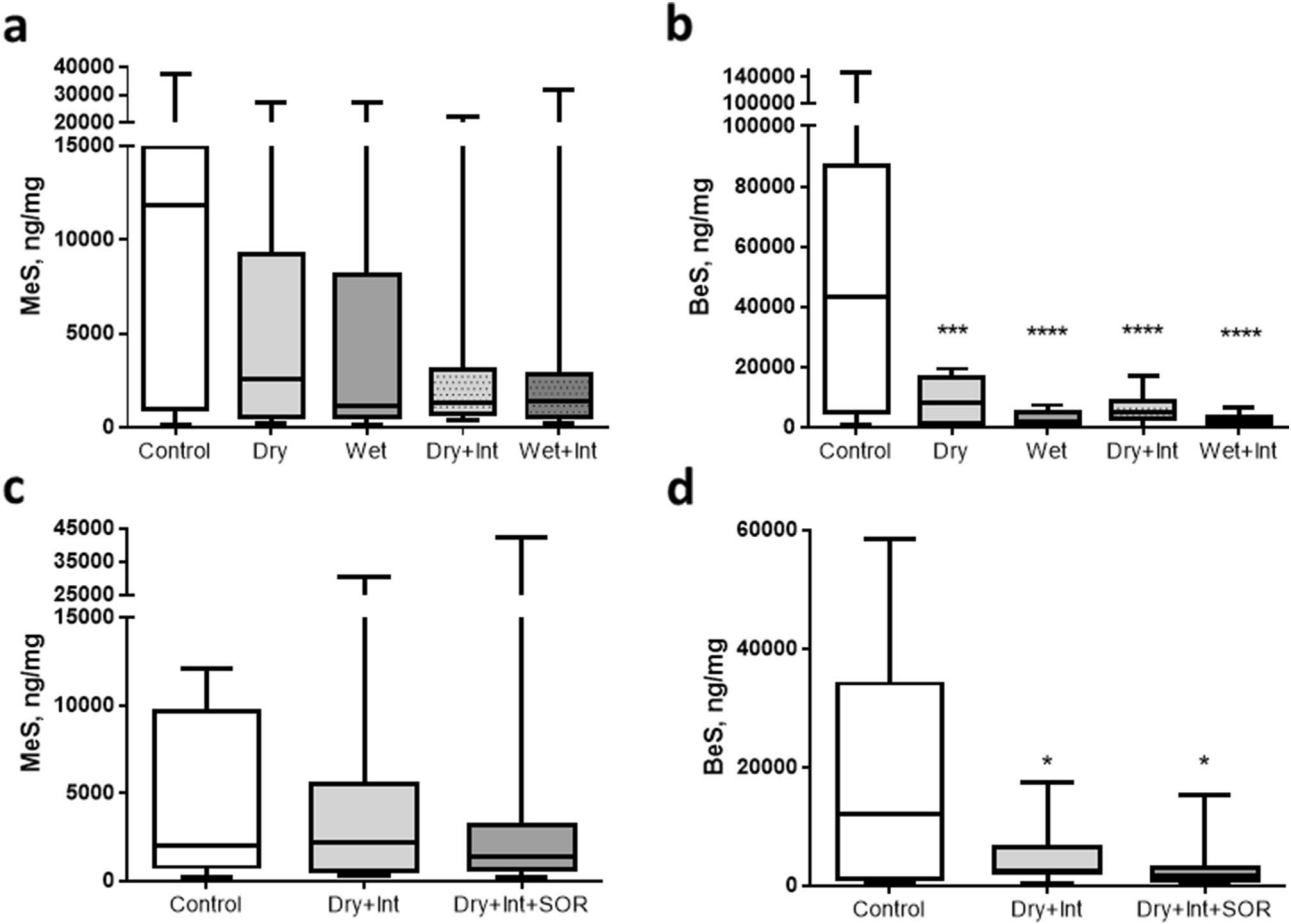
Recovery of MeS and BeS from hair for Study 1 (**a** and **b**) and Study 2 (**c** and **d**). Graphs show the median and inter–quartile range. Bars represent the minimum and maximum values. *, **, **** represent significance levels of < 0.05, < 0.01 and < 0.0001, respectively. Int = interim decontamination, SOR = mass decontamination.

MeS and BeS were detected above baseline levels in all hair samples from control and decontamination interventions (Table 1). Overall decontamination interventions resulted in significantly lower recoveries of MeS (F(1,11) = 7.09, *p* = 0.022) and BeS (F(1,11) = 11.76, *p* = 0.006) from hair compared to no-intervention controls (Fig. 1). Although for MeS, pairwise comparisons found that only the improvised wet condition was significantly lower than the control (*p* = 0.003), planned contrasts did not find any significant differences between the different active decontamination conditions (all ps > 0.10). Pairwise comparisons confirmed that the recovery of BeS from hair was significantly lower in each of the separate decontamination conditions compared to control (all ps < 0.01). Significantly less BeS was recovered in the improvised wet compared to improvised dry conditions (F(1,11) = 11.87, *p* = 0.005) (Fig. 1). There was no significant effect of decontamination stage (F(1,11) = 2.92, *p* = 0.116), or significant interaction between decontamination stage and type of improvised decontamination condition (F(1,11) = 1.99, *p* = 0.186).

**Table 1:** Recovery of MeS and BeS from the hair of participants in study 1 and study 2.

	Study 1		Study 2	
	MeS (ng/mg hair) Median (*range*)	BeS (ng/mg hair) Median (*range*)	MeS (ng/mg hair) Median (*range*)	BeS (ng/mg hair) Median (*range*)
Baseline	21.6 (*2.5–161.8*)	146.9 (*0.96–2695.7*)	40 (*3.6–512*)	198.1 (*115–2736.4*)
No intervention controls	11,892.1 (*180.4–37,653.5*)	43,234.3 (*1280.9–147,015.7*)	2191.2 (*228.3–12,112.6*)	6507.9 (*544.8–58,596.7*)
Decontamination interventions	1397.7 (*129.5–32,065.4*)	3958.5 (*723.2–19,644.8*)	1476.5 (*255.9–42,744.8*)	2608.2 (*399.1–18,168.7*)

**Figure 2:**
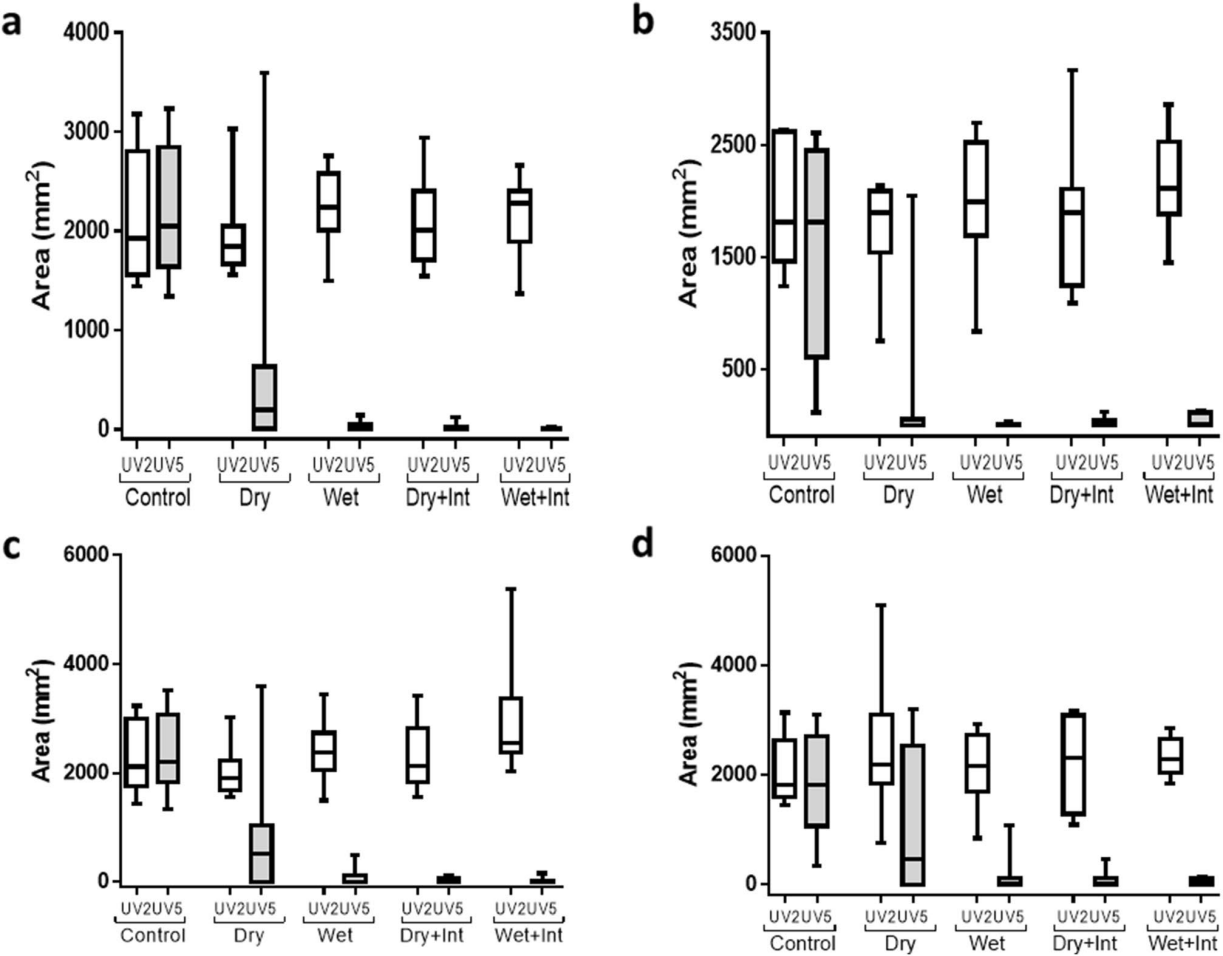
Surface area of simulants MeS and BeS on volunteer’s hair following application (UV2) and decontamination intervention (UV5/7) for each decontamination condition for Study 1 (**a** and **b**) and study 2 (**c** and **d**). Surface area was determined by calibrated UV photography and image analysis. Graphs show the median and inter–quartile range. Bars represent the minimum and maximum values. Int = interim decontamination.

The reduction in MeS and BeS recovery from hair following decontamination compared to no decontamination controls is supported by UV imaging. Post-decontamination images (UV5), showed a significant main-effect of decontamination condition on area of fluorescence (F(4,28) = 17.85, *p* < 0.001) (Fig. 2). Area of fluorescence was also significantly lower overall following decontamination compared to no decontamination control (F(1,7) = 39.26, *p* < 0.001), and pairwise comparisons confirmed that area was significantly lower in each of the separate active decontamination conditions compared to control (all *p*s < 0.05). Planned contrasts did not find any significant differences between the different active decontamination conditions (all *p*s > 0.10). Consistent with the BeS recovery data, the area of fluorescence was significantly lower overall for BeS (green) than MeS (red) (F(1,7) = 17.26, *p* = 0.004), and there was a significant interaction between colour and decontamination condition (F(1,7) = 5.23, *p* = 0.026). Separate analyses for each colour showed a significant main-effect of condition for both colours (both *p*s < 0.001), with significantly lower area following decontamination compared to no decontamination control (both *p*s < 0.01). Planned contrasts did not find any significant differences between the different active decontamination conditions for either colour (all *p*s > 0.10). All controls showed no cross-contamination.

#### Simulant deposition on the scalp

**Figure 3:**
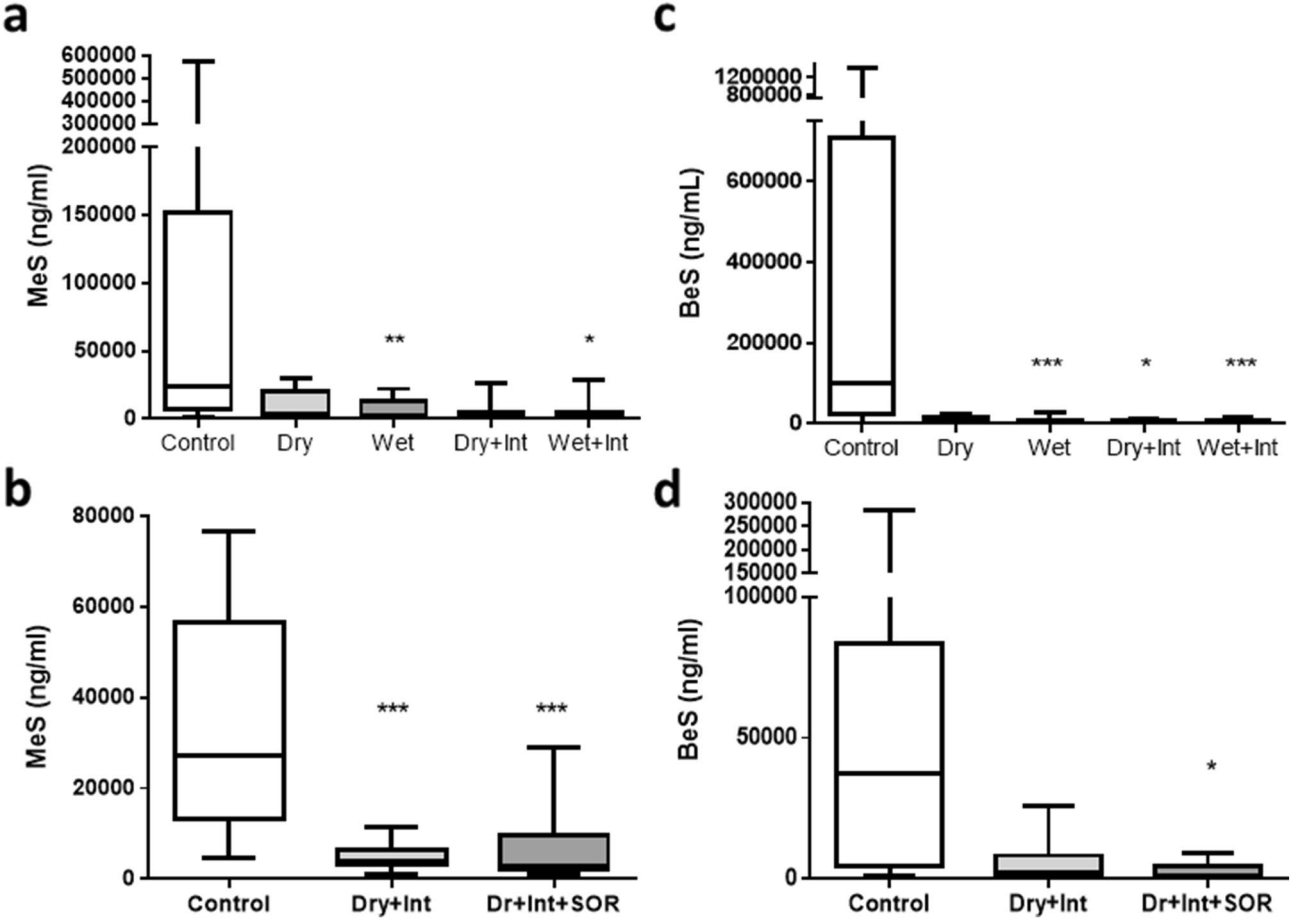
Recovery of MeS and BeS from scalp swabs Study 1 (**a** and **b**) and Study 2 (**c** and **d**). Graphs show the median and inter–quartile range. Bars represent the minimum and maximum values. *, **, *** represent significance levels of < 0.05, < 0.01 and < 0.001, respectively. Dr = dry decontamination, Int = interim decontamination, SOR = mass decontamination.

Swabs from the control condition showed deposition of both MeS and BeS on the scalp (Fig. 3). Post decontamination scalp swabs showed trends towards a significant main-effect of decontamination condition on the recovery of MeS (F(4,44) = 4.20, *p* = 0.064) and towards significantly less MeS recovered following decontamination compared to no decontamination control (F(1,11) = 4.25, *p* = 0.064). Pairwise comparisons found that this trend was maintained for each of the separate active decontamination conditions (all ps < 0.10) but planned contrasts did not find any significant differences between the different active decontamination conditions (all ps > 0.10).

Similarly, for BeS, there was significantly lower recovery in scalp swabs following decontamination compared to no decontamination control (F(1,11) = 6.24, *p* = 0.030) (Fig. 3) and pairwise comparisons confirmed that the recovery of BeS was significantly lower in each of the separate active decontamination conditions compared to control (all ps < 0.05). There was also a significant effect of stage of decontamination, with significantly less BeS recovered in the interim compared to improvised only conditions (F(1,11) = 5.96, *p* = 0.033). There was no significant effect of type of improvised decontamination (F(1,11) = 1.85, *p* = 0.202), or interaction between decontamination stage and type of improvised decontamination (F(1,11) = 0.56, *p* = 0.472).

#### Correlations between dry decontamination variables and decontamination efficacy

Participants spent between one minute four seconds and three minutes decontaminating in the dry decontamination condition (Table S1). More time spent decontaminating was associated with significantly lower recovery of both MeS and BeS in hair samples (r_s_ = − 0.61, *p* = 0.034 for MeS; r_s_ = − 0.77, *p* = 0.004 for BeS) however, there was no significant association between time spent decontaminating and recovery of either BeS or MeS in scalp swabs (both *p* > 0.10).

Although participants used between three and twelve sheets of white roll to perform dry decontamination, there was no significant association between number of sheets of white roll used (Table S1) and MeS or BeS recovered in either hair samples or scalp swabs (all *p* > 0.05).

#### Correlations between hair length and simulant recovery

Within the control condition, there was a strong correlation between hair length and simulant recovery in hair, with longer hair associated with lower recovery of both MeS and BeS (r_s_ = − 0.82, *p* = 0.002 for MeS; r_s_ = − 0.74, *p* = 0.010 for BeS). Longer hair was also associated with significantly lower simulant recovery in scalp swabs (r_s_ = − 0.63, *p* = 0.038 for MeS; r_s_ = − 0.77, *p* = 0.006 for BeS).

#### Systemic exposure to MeS and BeS

**Figure 4:**
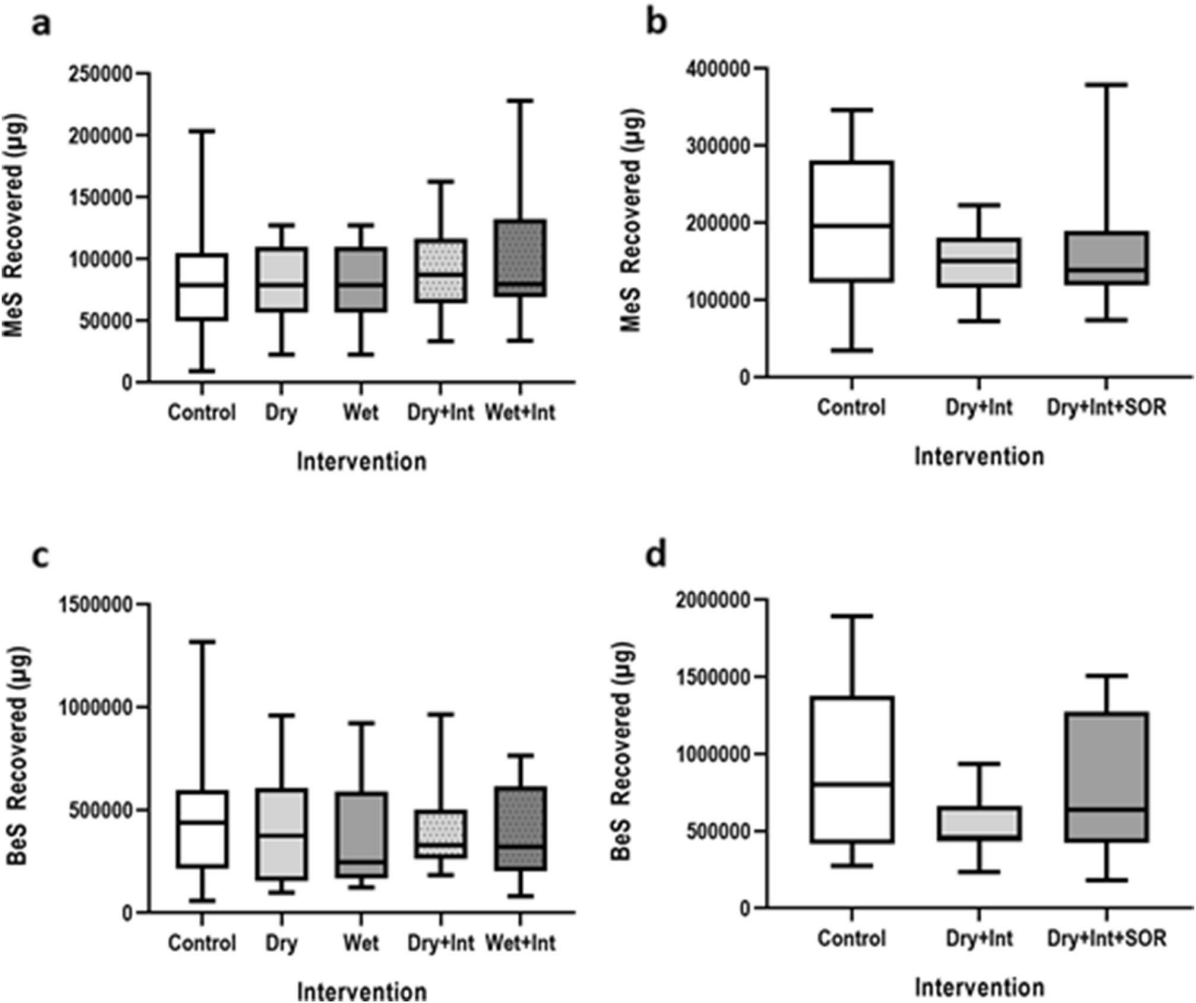
Methyl salicylate (MeS) detected in 24-urine samples from study 1 (**a**) and study 2 (**b**) and benzyl salicylate (BeS) detected in 24-urine samples from study 1 (**c**) and study 2 (**d**). Graphs show the median and inter–quartile range. Bars represent the minimum and maximum values. Int = interim decontamination, SOR = mass decontamination.

MeS and BeS were detected in the urine of all participants under all conditions, above baseline levels (Table 2). However, there was no significant difference between decontamination conditions for MeS or BeS excreted in baseline samples (F(4,44) = 1.54, *p* = 0.212 and F(4,44) = 0.77, *p* = 0.532, respectively), nor was there a significant main-effect of decontamination condition on MeS or BeS excreted in 24-h urine samples (F(4,44) = 1.53, *p* = 0.219) and F(4,44) = 0.40, *p* = 0.759, respectively) (Fig. 4). Planned contrasts found no significant difference between the active decontamination conditions and no decontamination controls (F(1,11) = 0.09, *p* = 0.768 for MeS and (F(1,11) = 0.53, *p* = 0.483 for BeS), or between the different active decontamination conditions (all ps > 0.05 for MeS and > 0.10 for BeS) (Fig. 4).

**Table 2:** Total excreted MeS and BeS detected in the urine of participants in study 1 and study 2.

	Study 1		Study 2	
	MeS (µg excreted) Median (*range*)	BeS (µg excreted) Median (*range*)	MeS (µg excreted) Median (*range*)	BeS (µg excreted) Median (*range*)
Baseline	26.7 (*2.8–186.4*)	21.61 (*9.5–1147.3*)	210.19 (*13.2–1126.2*)	310.7 (*24.2–2931*)
No intervention controls	78,347.2 (*8804.5–203,253.3*)	439,068.9 (*58,006.2–1,316,624*)	200,064.1 (*34,469.1–3,702,600.9*)	802,352.1 (*273,181.3–1,891,496.1*)
Decontamination interventions	79,415.3 (*13,134.3–227,527*)	322,095.3 (*78,807.8–963,476.7*)	138,122.3 (*72,250.1–378,320.2*)	529,770.3 (*179,142.8–1,506,445.7*)

### Study 2

Video reviews and detailed observations by the study staff confirmed that all participants adhered to the protocols as described.

MeS and BeS were detected above baseline levels in all hair samples from control and decontamination interventions (Table 1). Similarly, to Study 1 there was no main-effect of decontamination condition on MeS recovered in post-decontamination hair samples (F(2,20) = 0.06, *p* = 0.926), and no significant difference between the decontamination conditions and no decontamination control (F(1,10) = 0.14, *p* = 0.717) (Fig. 1). Conversely, significantly less BeS was recovered following decontamination compared to no decontamination control (F(1,10) = 5.73, *p* = 0.038), and pairwise comparisons confirmed that recovery was lower in both decontamination conditions compared to control (both ps < 0.05). There was also a trend towards significantly lower recovery following mass compared to interim decontamination (F(1,10) = 3.38, *p* = 0.096).

These data are supported by UV imaging (UV7) which showed significantly lower fluorescence overall following decontamination compared to no decontamination control (F(1,5) = 58.68, *p* = 0.001) (Fig. 2), and pairwise comparisons confirmed that fluorescent area was significantly lower in both active decontamination conditions compared to control (both ps = 0.001). There was no significant difference in area between the interim and specialist conditions (F(1,5) = 0.20, *p* = 0.675). In addition, there was no significant main-effect of colour (F(1,5) = 2.46, *p* = 0.177), or interaction between colour and decontamination condition (F(2,10) = 2.52, *p* = 0.173).

#### Simulant deposition on the scalp

Swabs from the control condition showed deposition of both MeS and BeS on the scalp (Fig. 3). Post decontamination scalp swabbing showed significantly lower MeS and BeS recovered following decontamination compared to no decontamination control (F(1,10) = 14.91, *p* = 0.003 and F(1,10) = 6.32, *p* = 0.031, respectively) and pairwise comparisons confirmed that recovery of both MeS and BeS was lower in both separate active decontamination conditions compared to controls (all ps < 0.05). For MeS, there was no significant difference in recovery between the interim and specialist conditions (F(1,10) = 0.68, *p* = 0.428) however, for BeS there was a trend towards significantly lower recovery following mass compared to interim decontamination (F(1,10) = 3.37, *p* = 0.096).

#### Correlations between hair length and simulant recovery

Within the control condition longer hair was significantly associated with lower recovery of both MeS and BeS in hair samples (r_s_ = − 0.78, *p* = 0.007 for MeS; r_s_ = − 0.95, p < 0.001 for BeS). There was no significant relationship between hair length and simulant recovery in scalp swabs (r_s_ = − 0.61, *p* = 0.062 for MeS; r_s_ = − 0.49, *p* = 0.154 for BeS).

#### Systemic exposure to MeS and BeS

In agreement with study 1, MeS and BeS were detected in all baseline samples (Table 2). There was no significant main-effect of decontamination condition on MeS excreted in 24-h samples (F(2,20) = 1.30, *p* = 0.282). Planned contrasts found no significant difference between the active decontamination conditions and the no decontamination control (F(1,10) = 1.30, *p* = 0.282) or between the interim and specialist condition (F(1,10) = 1.28, *p* = 0.284) (Fig. 4). However, one sample in the control condition was an extreme outlier, with a value approximately 3 standard deviations from the mean (Table 2). The participant also had high levels of MeS in both baseline and 24-h samples in all conditions, with 4 samples more than 2 standard deviations from the mean. Analysis for MeS in urine was repeated with this participant removed, and all effects remained non-significant (all *p* > 0.05). Whilst a general trend towards a significant main-effect of decontamination condition on BeS excreted in 24-h samples was detected (F(2,20) = 2.97, *p* = 0.082), planned contrasts found no significant difference between the active decontamination conditions and no decontamination control (F(1,10) = 1.96, *p* = 0.192), although there was significantly less BeS excreted in the interim condition compared to the specialist condition (F(1,10) = 6.28, *p* = 0.031).

## Discussion

This paper describes the first study on the efficacy of UK improvised, interim and specialist forms of mass casualty decontamination performed in sequence for the removal of two chemical simulants from the hair of human volunteers.

All decontamination interventions were shown to partially remove both MeS and BeS from the hair and scalp of participants but to varying extents. Dry decontamination alone, the current default method used in the UK as part of the Initial Operational Response (IOR) was shown to reduce the amount of MeS and BeS remaining on the hair of participants in study 1. Although results were only significant for the removal of BeS, these data are encouraging and suggest that dry decontamination, which has the advantage of being able to be instigated rapidly using any absorbent materials is beneficial and should remain the default option for casualty decontamination. Furthermore, this study suggests that the duration of dry decontamination is more important than the amount of dry decontamination material used, therefore in an actual incident it may be prudent for casualties to continue or repeat dry decontamination until the initiation of interim or mass decontamination procedures. Improvised wet decontamination (the rinse-wipe-rinse method) was shown to be more efficacious than dry decontamination but only for BeS. However, anecdotal feedback from study participants suggested that this method was difficult to perform and given the resources required for improvised wet decontamination (large amounts of water, vessels, flannels or sponges and detergent) it is unlikely to be a viable option if large numbers of casualties are requiring decontamination. For this reason, combined with feedback from the UK responder community, improvised wet decontamination was not examined in Study 2.

Larner *et al*^29^ recently concluded that a ‘Triple Protocol’ of dry decontamination combined with Ladder Pipe and Technical Decontamination was significantly better at removing MeS from hair compared to any single method alone. Several methodological factors may have confounded data interpretation in the Larner et al. study including uncontrolled dosing of MeS to the head and the swabbing used to recover MeS from the hair which may have been affected by occlusion of the application site post decontamination (as was frequently observed in the present study). Furthermore, as acknowledged by the authors the decontamination interventions were performed at unrealistic timescales. Two major advantages of the present study were the implementation of decontamination interventions at timescales reflective of current UK response capability and the excising of hair during sampling, enabling the measurement of both MeS and BeS on the hair surface as well as MeS and BeS bound to hair. On closer examination of the Larner et al. data^29^ it appears that the ‘Triple Protocol’ was only significant compared to an untreated control group and no significance was reported for the ‘Triple Protocol’ vs dry decontamination, Ladder Pipe or Technical Decontamination performed alone. The present study agrees with this interpretation. Although the efficacy of some individual decontamination interventions has been demonstrated here (particular for BeS) there was limited evidence of the cumulative effect of decontamination interventions. Whilst it remains logical that a combination of dry and wet decontamination interventions performed in sequence would result in greater removal of chemical contamination, the present study and the Larner et al. study, may have been statistically underpowered to detect this effect. Further work is required to repeat these studies with larger groups of human volunteers.

Data showing significant removal of BeS from hair in both study 1 and study 2 is encouraging, particularly for a lipophilic simulant representative of persistent chemical threats such as Novichok. However, in contrast, whilst all decontamination interventions were shown to remove MeS from hair to equivalent degrees’, significance between control and decontamination was only reached for study 1. This could be due to increased variability in the recovery of MeS or possible loss of MeS from the control condition due to evaporation, both a consequence of the greater volatility of MeS compared to BeS. The approximate threefold higher recovery of BeS compared to MeS in the control condition potentially supports this assumption but it should also be noted that a higher dose of BeS (not diluted 1:1 with vegetable oil) was applied. Alternatively, increased spreading of MeS beyond the recovery areas was possible however, this is not supported by the UV imaging data. Instead, these data could suggest that MeS is more persistent in hair, either as a result of the greater lipophilic nature of the MeS and vegetable oil mixture or possibly due to MeS binding to hair more effectively than BeS. Indeed, several studies have indicated the potential for hair to bind chemical contaminants^17,23,24^. Further studies with a greater sample number are required to elucidate these findings.

Whilst it has previously been suggested that hair could act as a protective barrier for the scalp^7,30^, swabbing showed that both MeS and BeS penetrated to the scalp during both studies. Whilst this could be related to the method of simulant application certain chemicals have been demonstrated to diffuse rapidly through hair sebum to the follicles from which they can be absorbed^17,22^. However, all decontamination methods were highly effective at reducing the presence of simulant on the scalp. These data are consistent with the high efficacy observed for the same decontamination methods used on human skin^13^ however, it remains unclear if this observation was a direct result of removing simulant from the scalp skin or an indirect effect of removing simulant from the hair. Further studies are required to provide clarity but nonetheless this finding could have potential implications for the management of contaminated persons. Perhaps a viable emergency decontamination approach would be to remove as much simulant from the scalp (the site of greatest potential chemical absorption) to rapidly reduce systemic exposure and then manage the remaining chemically contaminated hair as a secondary measure.

For all interventions, substantial quantities of MeS and BeS remained in the hair following decontamination. This finding is consistent with previous in vitro studies where up to 50% of the tested chemical warfare agent (VX, sulphur mustard) or lipophilic chemical simulant (phorate or MeS) were found to be remaining in the hair following decontamination^16,19,31^. However, it is not consistent with a recent human volunteer field exercise from Chilcott *et al*^30^ that demonstrated high decontamination efficacy for MeS contaminated hair. These data however, should be treated with caution as the recovery of MeS in volunteers (including no-decontamination controls) across all application sites was low and highly variable. This could be due to methods of sampling (hair was not excised) or reflective of the challenges associated with using simulants under field exercise conditions. Interpretation of hair decontamination is complicated by the high variabilities in recovered simulant concentration as we also observed in this study. Although Chilcott et al. took steps to reduce confounds such as standardised sample application, application site templating and multiple sampling, there are other factors (e.g. volunteer movements/interactions, hair movement, fluctuations in weather conditions etc.) that are more difficult to control for in a volunteer study without taking large amounts of hair samples.

This study was the first to examine intact simulants in the urine of volunteers as a measure of systemic exposure in a study of hair decontamination. Previous studies attempting to analyse MeS metabolites in urine as a measure of decontamination efficacy^29^ have failed possibly due to extraneous and uncontrolled sources of salicylates e.g. foodstuffs. A subsequent study by James *et al*^32^ measuring parent MeS in urine also suggested that exogenous sources of MeS can confound data interpretation. Whilst the MeS data in this study should be interpreted within this context both MeS and BeS concentrations in this study were much higher in control and intervention samples than in baseline samples indicating that a systemic effect due to dermal penetration was being observed. Results from both study 1 and study 2 showed that despite hair and scalp decontamination at realistic timescales, none of the interventions (including those performed in sequence) had a significant effect on the total recovery of BeS or MeS detected in urine over a 24-h period. This observation suggests that the bioavailable dose of MeS and BeS is entering the body prior to the dry decontamination intervention at 15 min, raising important considerations with respect to the speed of decontamination.

These findings raise important questions regarding the clinical management of contaminated hair. Contaminated hair is not only a direct secondary contamination risk to first responders, but also hospital receivers, the public or the exposed person. Studies by Spiandore et al. (39) demonstrated that MeS and another sulphur mustard simulant chloroethyl ethyl sulphide trapped in hair could rapidly desorb into the surrounding atmosphere and therefore pose a substantial inhalation risk. The removal of contaminated hair may need to be considered post decontamination, however this is likely to be a clinical decision informed by the nature of the contaminant (if known), the extent of contamination, and the clinical condition of the exposed person. Furthermore, the additional burden created by the removal of hair during a mass casualty incident would need careful consideration. Further research is therefore required to elucidate the fate of chemical contaminants bound to hair, and the risks (e.g. off-gassing, surface transfer) they could pose to others particularly if decontamination is not fully effective.

This study does have limitations. First, only two chemical simulants were evaluated, albeit with differing physicochemical properties. Whilst this is an improvement on previous studies, caution is advised when extrapolating results from this study to other types of chemicals. Further work should examine the efficacy of decontamination interventions on a broader range of chemical simulants, bracketing the physicochemical properties of agents of concern. Second, although the hair and scalp sampling methodology were an improvement on previous methods, they were semi-quantitative only and it was not possible to accurately calculate the total dose of simulant remaining on the hair or the scalp. Furthermore, the current study did not control for hair length and as shown here this can affect simulant recovery. The importance of controlling for hair length should be considered in any future similar studies. Finally, UV imaging of hair showed a consistent reduction in simulant fluorescent area following all decontamination interventions, however occlusion of the application site in long haired volunteers precluded UV imaging analysis. Although the hair sampling strategy was designed to limit the impact of this eventuality, it remains likely that some of the simulant dose may not have been visible by imaging, or available for sampling when hair was excised. Future studies could evaluate enhanced measures to ensure more reproducible recovery from hair including isolating small sections of hair for simulant application and targeted recovery. However, this would need to be balanced against the need to realistically reflect the conditions under which emergency decontamination is conducted; a strength of this study. Alternatively, hair fluorescence could be used to guide sample collection to the area of simulant application. However, a strategy for overcoming sample selection bias would need to be developed. Finally, the findings of this study are mainly applicable to casualties who are can self-decontaminate. Although some work has been conducted on decontamination efficacy for non-ambulant casualties^10^ further studies are required to develop optimal decontamination strategies for these and other vulnerable populations.

In conclusion, this study has demonstrated that current UK decontamination methods (including default dry decontamination) performed in sequence are partially effective at removing MeS and BeS from hair and underlying scalp. While the interventions tested during this study proved to be effective at reducing external contamination that may have otherwise spread to further casualties, the analysis of intact MeS and BeS in urine indicated that decontamination had no significant effect on systemic exposure, raising important considerations with respect to the speed of decontamination and the perceived reduction of risk to the casualty. Further work is required to repeat these experiments with a broader range of chemical simulants, a larger group of human volunteers and at different/shorter intervention times.

## Methods

### Study design and participants

Two within-subjects, studies were used to investigate the effects of improvised, interim and specialist mass-casualty decontamination protocols in sequence on the removal of two chemical simulants from the hair of human volunteers. Procedures were recorded by video to enable identification of any protocol deviations.

Study 1 examined five decontamination conditions whereas study 2 examined three (Table 3). A pragmatic decision not to include improvised wet decontamination in study 2 was made following feedback from UK First Responders that dry decontamination would be the default initial decontamination step for hair during a chemical incident.

**Table 3:** Decontamination conditions used in each study.

Decontamination condition	Study 1	Study 2
No decontamination (control)	Yes	Yes
Improvised dry (dry)	Yes	No
Improvised wet (wet)	Yes	No
Dry + interim	Yes	Yes
Wet + interim	Yes	No
Dry + interim + specialist mass (SOR)	No	Yes

A power calculation using data from a previous trial of improvised and interim decontamination protocols on the removal of a chemical simulant from the skin of human volunteers^13^, determined that a sample size of 12 was sufficient to detect effects under the experimental conditions (power = 0.823). The sample size also allowed contingency in the event of drop-outs or missing data. Volunteer recruitment criteria, methodology and screening were as previously described^13^. Twelve adults (4 female, 8 male) participated in Study 1. Owing to one drop-out eleven adults (6 female, 5 male) participated in Study 2.

Both studies were conducted in according to the principles of the Declaration of Helsinki. Ethical approval was independently granted by Public Health England’s Research and Governance Group. All participants gave informed consent to taking part in the studies.

Participants completed all decontamination conditions for both studies in a randomised order during separate study sessions. Each study session was separated by a minimum of one week.

Participants were asked not to use any products (including cosmetics) containing MeS or BeS or wash their hair for 24 h prior to and following the study. They were also instructed to avoid the consumption of certain foods suspected to contain MeS according to a previous study^13^.

### Decontamination conditions

*Control (C):* Participants did not undergo any form of decontamination. Participants were asked to stand for the duration of the study and were moved to the different, pre-defined decontamination areas to replicate the movement of volunteers’ in the other decontamination conditions.

*Improvised dry decontamination (dry):* Improvised dry decontamination was conducted according to current UK Initial operational response guidance^33^. Improvised dry decontamination consisted of removal of the chemical simulant from hair using 1-ply sheets of white roll folded in half twice. Participants had three minutes to complete the process using as many sheets of white roll as they saw fit but only using one sheet at a time. If participants felt like they had finished decontaminating before the full three minutes had elapsed they were able to stop decontaminating. The researcher informed the volunteer when each minute had elapsed.

*Improvised wet decontamination (wet):* Improvised wet decontamination was conducted according to current UK Initial operational response guidance^33^*.* Two buckets containing 5L of room temperature water (mean water temperature = 22.8 °C) and one bucket containing a 5L solution of room temperature water with 0.5% Fairy washing up liquid detergent (Procter and Gamble, UK) were provided. Participants had three minutes in total to complete a rinse-wipe-rinse procedure^33^ as follows:

- One minute to rinse the hair with water using a 1 L jug;
- One minute to wipe the hair with a sponge and the detergent water;
- One minute to rinse the hair for a second time using a 1 L jug.

Participants were free to rinse and wipe as many times as they felt necessary during the one minute periods.

*Interim decontamination*: Interim decontamination involved the participants walking through a ‘ladder-pipe’ shower system set up by trained UK Fire and Rescue Service staff and designed to follow a typical operational protocol for interim decontamination. The system consisted of four hose reels and branches from a fire tender suspended from two ladders to create two showering positions (two branches per position). Water was delivered from a storage tank through the fire tender at approximately 20 °C. . The participants spent a total of 90 s in the shower system during which they were instructed to spend 45 s actively washing their hair at the first shower position and then 45 s actively washing their whole body using their hands at the second shower position.

*Specialist mass decontamination (SOR):* Specialist decontamination involved the use of a mass decontamination unit (MDU-MD1) set up by trained UK Fire and Rescue Service staff. For this study, the disrobe and re-robe sections of the MD1 were removed leaving only the showering section. Water at an average temperature of 28.1 °C (pressure of 2.5–3 bar, equating to a flow of ~ 10 L min-1 per person) and detergent (HOSPEC Concentrated General Purpose liquid detergent, McBride, Manchester, UK) was supplied to the unit using a water boiler (Professional Protection Systems, UK) with integrated Dosatron. The boiler automatically controlled the three-minute wash cycle consisting of two minutes washing with detergent and one-minute rinsing without detergent. Participants were provided with a flannel (size and make) prior to entry and asked to stand in one of the pre-designated showering positions. Each showering position consisted of five spray nozzles (one to the front of the participant, two to the rear and two above). An electronic audio-visual entry system integral to the MD1 informed participants when to enter and exit the showering section.

Participants walked towards a pre-marked shower position and conducted a full body decontamination, using a flannel, for three minutes (two minutes with a 0.5% (v/v) detergent solution and one minute with clean water).

Participants commenced each decontamination condition at pre-set times to replicate the earliest expected time that such decontamination procedures would be operational during a real chemical incident (dry/wet at 15 min, interim at 25 min and mass at 60 min).

#### Procedure

**Figure 5:**
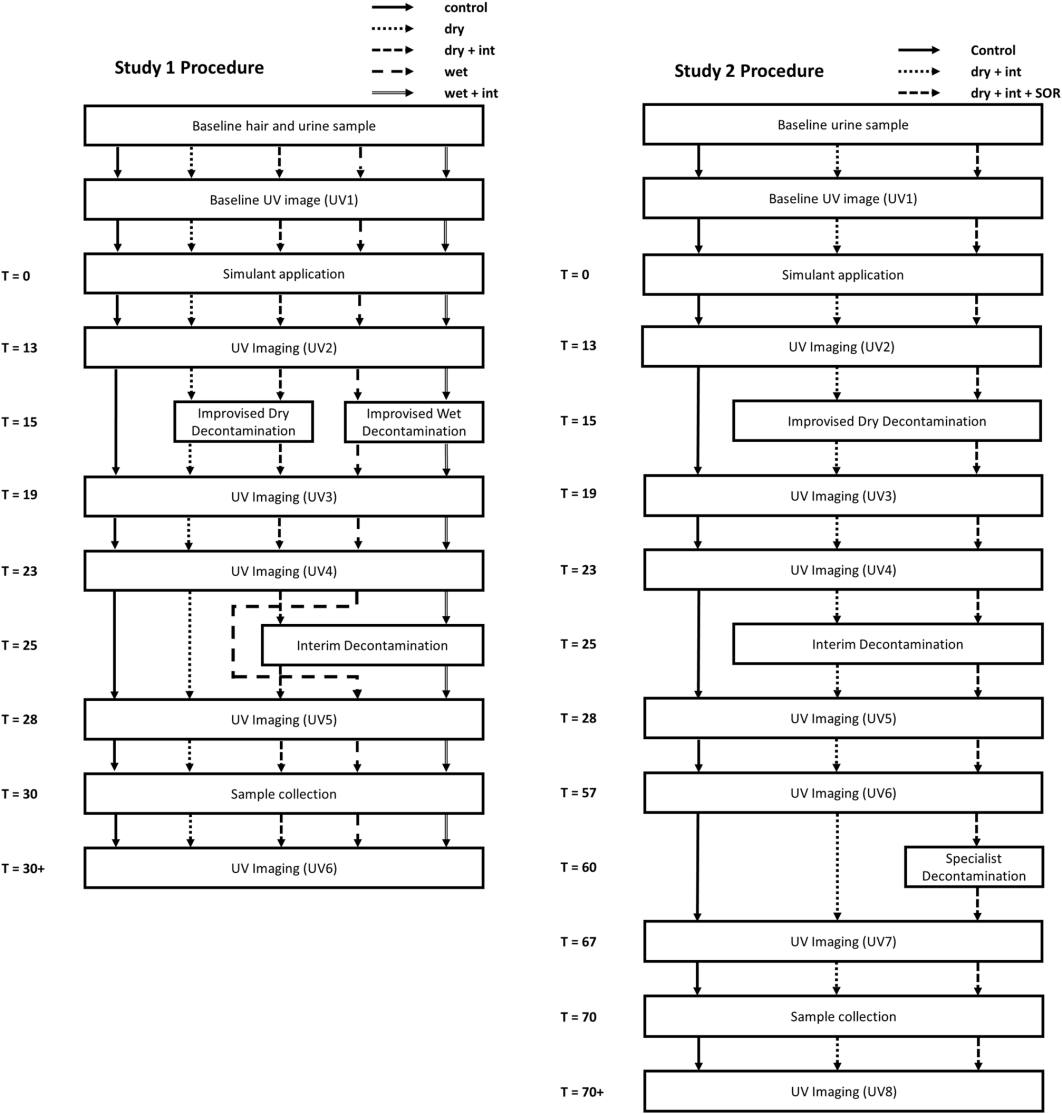
Schematic representation of the protocols for study 1 and 2 showing study conditions and the timings of the decontamination interventions and UV imaging.

Figure 5 shows the detailed timelines for study 1 and 2, respectively. Descriptive statistics for the study variables are presented in supplementary material (Tables S1 and S2).

*Baseline sampling and imaging:* A baseline urine sample (10–50 ml) was collected as previously described^13^. Participants were asked to disrobe and change into black or dark blue polyester/nylon swimwear and black shoes provided. A baseline sample of hair was then collected from each volunteer (negative control) prior to simulant application. Approximately 5–10 hairs were cut from the posterior vertex of the head using scissors and were placed into a pre-weighed vial with 10 ml dichloromethane (DCM). A baseline UV image of the back of the participant’s head was collected prior to simulant application (UV1).

*Simulant application:* Two chemical simulants were applied directly to the hair of participants. Simulant one consisted of a 1:1 mix of MeS (99.9%, Fisher Scientific, UK) and vegetable oil (The Cooperative, UK, added to increase persistence)^13^ and 4 mg/ml Invisible Red S (IRS, Chemox Pound Ltd.). Simulant two consisted of benzyl salicylate (99.9%, Fisher Scientific, UK) with 4 mg/ml Invisible Green S (IGS).

**Figure 6:**
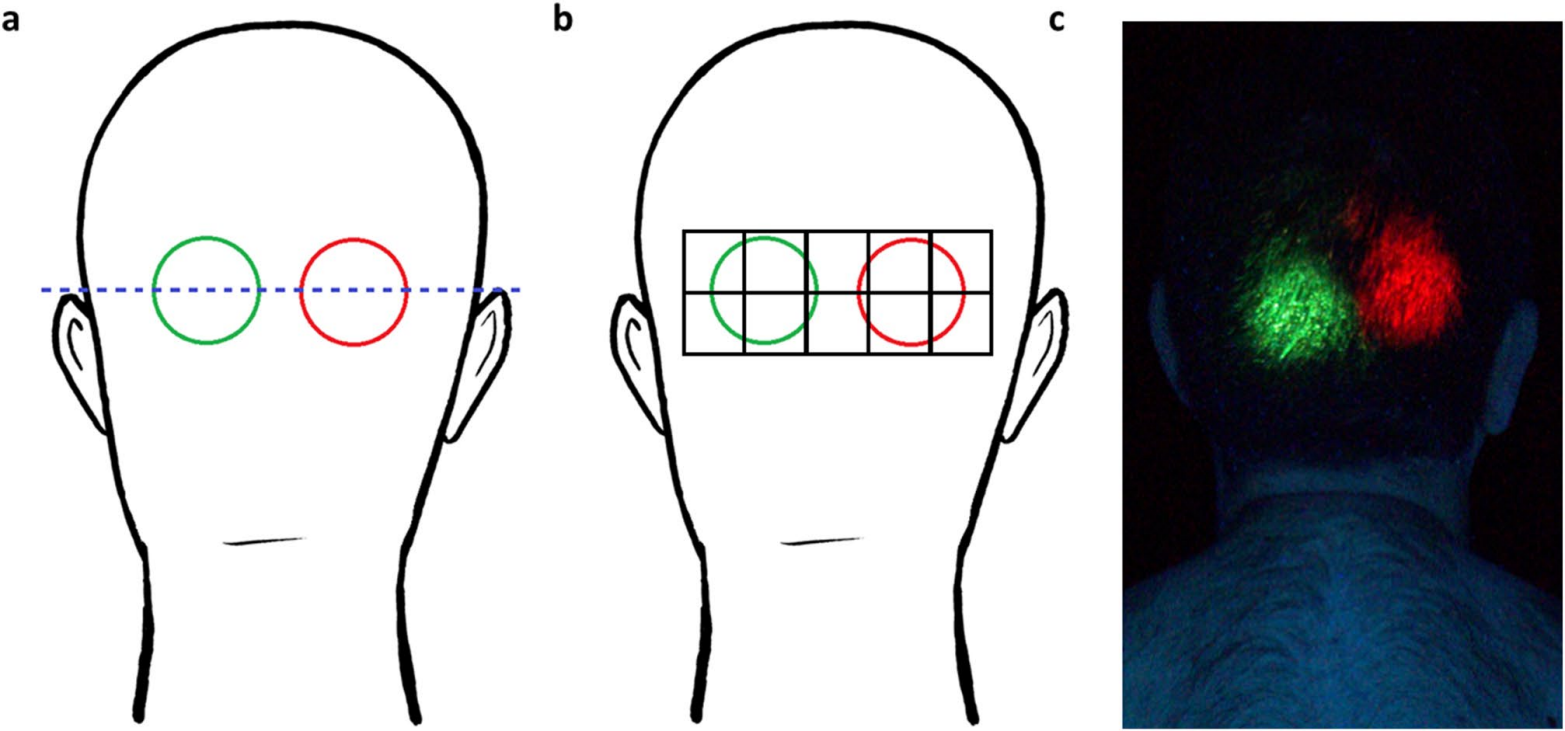
(**a**) Schematic representation of the simulant application zones on the hair of participants, MeS (red circle) and BeS (green circle). (**b**) Schematic representation of the hair sampling area showing division of the application zones. (**c**) Example application of MeS and BeS on a participant’s head. Photographed under UV light.

Both simulants (500 µl) were applied to the analytical application zone on the back of the head (Time zero, T = 0), in line with the top of the volunteer’s ears (Fig. 6) using two Badger Renegade Series Krome Gravity Feed airbrushes mounted to a custom adjustable rig (Supplementary Fig. S1) designed to ensure the distance between the airbrushes and the application sites (10 cm) remained consistent across participants. A Revell Master Class Compressor (Wonderlandmodels.com, UK) supplied compressed air to the brushes at 65 psi. The airbrushes were set to maximum spray velocity at a pressure of 10 psi. Simulant application occurred in an enclosed tent. Participants were provided with goggles, a nose clip and respiratory protection (mouthpiece connected to an external fresh air supply). Participants only wore respiratory protection for simulant application and were removed from the application area immediately post-application.

Measurements of simulant surface area by UV photography post-simulant application (UV 2) were consistent between different decontamination conditions and colours. There was no significant main effect of decontamination condition on area of fluorescence, no significant main effect of colour and no significant interaction between colour and decontamination condition for both studies (all ps =  > 0.20) confirming reproducibility of the application method.

*Decontamination:* Following simulant application, participants were reminded to avoid touching their hair to prevent transfer of simulant to other parts of the body. UV images were taken at set time points pre-and post-simulant application and pre-and post-decontamination (Fig. 5). As well as determining the area of fluorescent contamination the images also served as a quality control to monitor for inadvertent transfer of simulant from the hair to other parts of the body. At 15 min, participants were asked to stand in a pre-defined position ready to undertake the first decontamination protocol (conditions dry and wet). For conditions involving interim decontamination (dry + interim, wet + interim and dry + interim + SOR), participants entered the shower corridor at 25 min where they completed the interim decontamination protocol). For conditions not requiring interim decontamination (control, dry and wet), participants walked through the shower corridor but did not undergo any decontamination.

In Study 1, following the penultimate UV image (UV5), participants sat on a stool for sample collection at 30 min. In Study 2, following UV5 there was a 29-min wait period before mass decontamination in the MDU commenced at 60 min (dry + interim + SOR). For conditions not requiring mass decontamination in the MDU (control, dry + interim), participants stood in the interim shower corridor but did not undergo any decontamination. Following the penultimate UV image (UV7), participants sat on a stool for sample collection at 70 min.

At the end of all sessions, participants were provided with a towel and changed back into their clothing. Participants were instructed not to wash their hair or use any hair products until the end of urine collection (> 24 h).

*Hair and urine sample collection:* For hair sample collection, the application zones on the participants’ head were visually divided into 10 equal areas across 2 rows covering both simulant application zones. Eight to ten hairs were cut from the root from each of the 20 areas and placed into a pre-weighed air-tight vial containing 10 ml DCM.

To assess simulant deposition on the scalp, scalp swabs were taken from the centre of both the MeS and BeS application sites using cotton swabs (Johnson’s, UK). The hair was carefully parted and the scalp was swabbed three times in an upwards motion covering an area of approximately 1.25 cm^2^. Both swabs were placed into a single air-tight vial containing 10 ml DCM.

Participants were instructed to collect their urine in one combined sample in a 4L container for the 24 h following simulant application, ensuring their last sample was as close to 24 h’ as possible. Aliquots (50 ml) of all samples were stored in a − 20 °C Human Tissue Authority (HTA) licenced freezer prior to analysis.

### UV photography and image analysis

UV photography was conducted in a bespoke Mobile Image Analysis Unit (MIAU) as previously described^13^ with the following modifications. Instead of standing, participants sat with their backs facing the camera, on a stool, placed against a pre-positioned wooden marker to ensure consistent positioning. To aid analysis of fluorescent simulant area (cm^2^) calibration discs were created consisting of MeS with IRS simulant and BeS with IGS applied to a Whatman No.1 110 mm Filter Paper discs (Scientific Laboratory Supplies, Nottingham, UK) so that the whole area of the filter paper was covered completely in simulant. A calibration disc was placed adjacent to the head of the volunteer and was routinely changed throughout the study. Image files were analysed using bespoke software^13^. Image files were segmented to extract clusters of 20 or more red or green pixels and the number of red or green pixels and total intensity of pixel was recorded for each cluster. Area of fluorescence was calculated by comparing the number of pixels in each cluster against the number of pixels for the area calibration disc. Area fluorescence was only analysed for participants with short hair owing to occlusion of the application site following decontamination for long-haired participants.

### Hair, scalp swab and urine sample analysis

MeS and BeS in hair samples and swabs were measured by gas chromatography triple quadruple mass spectrometry (GC–MS/MS) according to the method described by James *et al*^34,35^. MeS and BeS in 24-urine samples were measured by GC–MS/MS according to James *et al*^32^.

### Interpretation and statistics

Outcome measures were analysed using repeated measures ANOVA, with decontamination condition and colour for UV imaging (red, green) as an independent variable. For all outcome measures, Alpha was 0.05, with Huynh–Feldt sphericity corrections applied for repeated measures effects. Analyses were conducted using IBM SPSS Statistics v.25.

In Study 1, planned contrasts compared: the four decontamination conditions compared to the control; the main effect of the decontamination stage (improvised dry/wet only compared to improvised wet/dry plus interim conditions); the main effect of type of improvised decontamination (dry vs wet conditions; dry, dry + interim vs. wet, wet + interim); and the interaction between the decontamination stage and type of improvised decontamination (dry, wet + interim vs. wet, dry + interim). For Study 2, planned contrasts compared: the two decontamination conditions compared to the control and the interim compared to mass decontamination conditions.

## Supplementary information

Supplementary information.

## Data Availability

The datasets generated during and/or analysed during the current study are not publicly available at present but are available from the corresponding author on reasonable request.
