# Author Correction: Human volunteer study of the decontamination of chemically contaminated hair and the consequences for systemic exposure

**DOI:** 10.1038/s41598-021-88761-z

**Published:** 2021-04-21

**Authors:** Samuel Collins, Thomas James, Felicity Southworth, Louise Davidson, Natalie Williams, Emily Orchard, Tim Marczylo, Richard Amlôt

**Affiliations:** 1 Chemical and Environmental Effects Department, Centre for Radiation, Chemicals and Environmental Hazards, Public Health England, Didcot, Oxfordshire UK; 2 Toxicology Department, Centre for Radiation, Chemical and Environmental Hazards, Public Health England, Didcot, Oxfordshire UK; 3 Behavioural Science Team, Emergency Response Department Science and Technology, Public Health England, Porton Down, UK

Correction to: *Scientific Reports* 10.1038/s41598-020-77930-1, published online 30 November 2020

This Article contains errors in Figure 2. The correct Figure 2 appears below as Figure 1.

**Figure 1:**
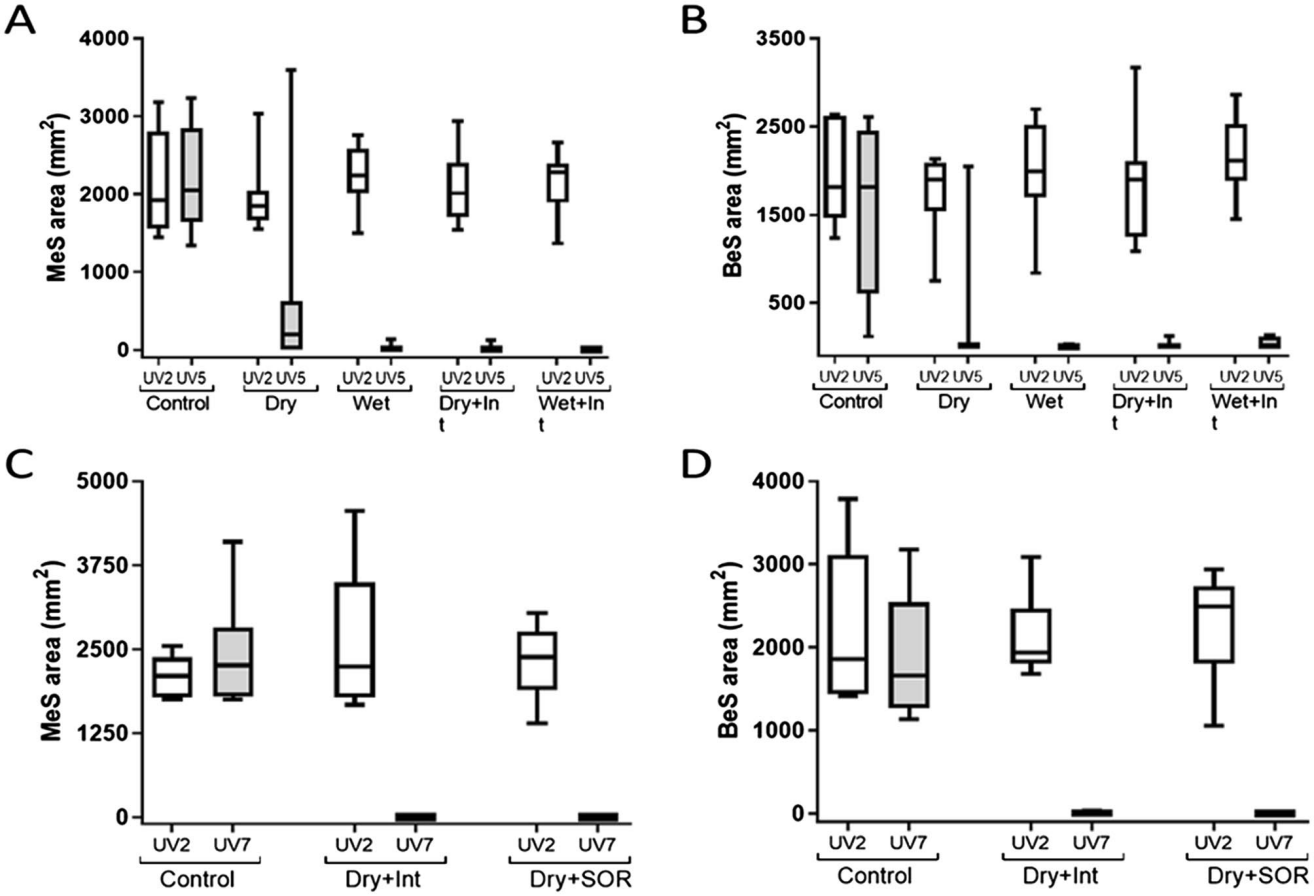
Surface area of simulants MeS and BeS on volunteer’s hair following application (UV2) and decontamination intervention (UV5/7) for each decontamination condition for Study 1 (**A** and **B**) and study 2 (**C** and **D**). Surface area was determined by calibrated UV photography and image analysis. Graphs show the median and inter–quartile range. Bars represent the minimum and maximum values. Int = interim decontamination.

