# Supplementary material for: Human volunteer study of the decontamination of chemically contaminated hair and the consequences for systemic exposure
Source: Sci Rep. 2020 Nov 30;10:20822. doi: 10.1038/s41598-020-77930-1 (PMC7705014; doi:10.1038/s41598-020-77930-1)
Supplement: Supplementary file 1 — Supplementary information. [file 41598_2020_77930_MOESM1_ESM.pdf]

Human volunteer study of the decontamination of chemically contaminated hair and the consequences for systemic exposure

Samuel Collins[1], Thomas James[1], Felicity Southworth[3,] Louise Davidson[3], Natalie Williams[3], Emily Orchard[3], Tim Marczylo[2], Richard Amlôt[3].

[1] Chemicals and Environmental Effects Department, Centre for Radiation, Chemicals and Environmental Hazards, Public Health England, Didcot, Oxfordshire, UK

[2] Toxicology Department, Centre for Radiation, Chemicals and Environmental Hazards, Public Health England, Didcot, Oxfordshire, UK

[3] Behavioural Science Team, Emergency Response Department Science & Technology, , Public Health England, Porton Down, UK

Supplementary materials

Supplementary Figure S1

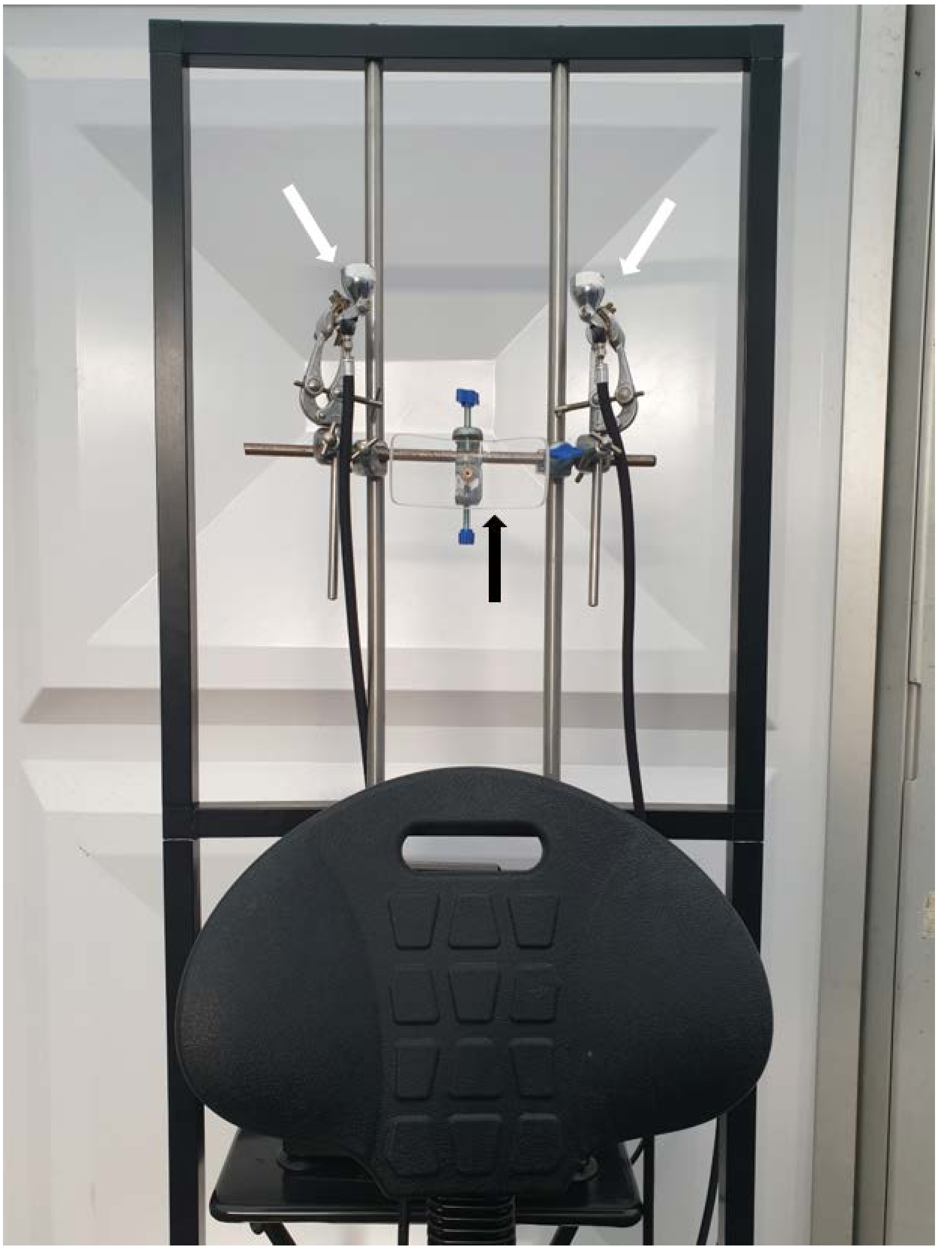

Figure S1: Custom adjustable simulant application rig with compressor (behind chair) and attached airbrushes (white arrows). Participants sat in the height adjustable chair with their neck against the acrylic plate (black arrow). To maintain a consistent distance from the back of the head to the air brushes 10cm spacers were attached to the end of the airbrushes and removed before application (not shown).

Supplementary Table S1. Mean (*SD*) and range for decontamination condition variables in Study 1.

	Control	Dry	Wet	Dry+Int	Wet+Int
Ambient temp. (°C)	23.4 (*2.66*), 19.6 – 26.6	23.0 (*2.66*), 18.4 – 26.5	23.4 (*1.82*), 20.7 – 26.9	23.3 (*2.20*), 18.2 – 26.6	23.2 (*2.95*), 17.7 – 26.8
Quantity of white roll (no. sheets)	-	6 (*2.63*), 3 - 12	-	5 (*2.15*), 3 - 9	-
Dry decontamination time (min:s)	-	2:27 (*0:44*), 1:04 – 3:00	-	2:27 (*0:51*), 1:00 – 3:00	-
RWR water temp. (°C)	-	-	22.8 (*1.79*), 19.4 – 26.0	-	23.0 (*1.97*), 19.6 – 26.3
Interim water temp. (°C)	-	-	-	19.4 (*0.78*), 18.0 – 20.5	19.6 (*1.97*), 17.4 – 24.5

Supplementary Table S2. Mean (*SD*) and range for decontamination condition variables in Study 2.

	Control	Dry+Int	Dry+Int+SOR
Ambient temp. (°C)	23.8 (*1.82*), 20.1 – 26.8	23.7 (*1.73*), 20.8 – 26.0	23.9 (*2.13*), 21.1 – 27.1
Quantity of white roll (no. sheets)	-	6 (*2.00*), 2 - 9	6 (*3.11*), 3 - 12
Dry decontamination time (min:s)	-	2:35 (*0:25*), 2:00 – 3:00	2:35 (*0:33*), 1:30 – 3:00
Interim water temp. (°C)	-	20.1 (*0.46*), 19.6 – 21.0	20.6 (*1.68*), 18.8 – 25.3
SOR water temp. (°C)	-	-	28.1 (*1.30*), 25.8 – 30.1
